# Genome-Wide Association Study of Clinical Outcome After Aneurysmal Subarachnoid Haemorrhage: Protocol

**DOI:** 10.1007/s12975-021-00978-2

**Published:** 2022-01-06

**Authors:** Ben Gaastra, Sheila Alexander, Mark K. Bakker, Hemant Bhagat, Philippe Bijlenga, Spiros Blackburn, Malie K. Collins, Sylvain Doré, Christoph Griessenauer, Philipp Hendrix, Eun Pyo Hong, Isabel C. Hostettler, Henry Houlden, Koji IIhara, Jin Pyeong Jeon, Bong Jun Kim, Munish Kumar, Sandrine Morel, Paul Nyquist, Dianxu Ren, Ynte M. Ruigrok, David Werring, Ian Galea, Diederik Bulters, Will Tapper

**Affiliations:** 1 Clinical Neurosciences, Clinical and Experimental Sciences, Faculty of Medicine, University of Southampton, Southampton, SO17 1BJ UK; 2 Department of Neurosurgery, Wessex Neurological Centre, University Hospital Southampton, Southampton, SO16 6YD UK; 3 School of Nursing, University of Pittsburgh, 3500 Victoria Street, Pittsburgh, PA 15261 USA; 4 Department of Neurology, University Medical Center Utrecht Brain Center, University Medical Center Utrecht, Heidelberlaan 100, 3584 CX Utrecht, the Netherlands; 5 Division of Neuroanaesthesia, Department of Anaesthesia and Intensive Care, Postgraduate Institute of Medical Education and Research (PGIMER), Chandigarh, India; 6 Neurosurgery Division, Department of Clinical Neurosciences, Faculty of Medicine, Geneva University Hospitals, Geneva, Switzerland; 7 University of Texas Houston Health Science Center, Houston, TX USA; 8 Geisinger Commonwealth School of Medicine, Scranton, PA USA; 9 Departments of Anesthesiology, Neurology, Psychiatry, Pharmaceutics, and Neuroscience, College of Medicine, Center for Translational Research in Neurodegenerative Disease, McKnight Brain Institute, University of Florida, Gainesville, FL USA; 10 Department of Neurosurgery, Geisinger, Danville, PA USA; 11 Department of Neurosurgery, Christian-Doppler Klinik, Paracelsus Medical University, Salzburg, Austria; 12 Department of Neurosurgery, Saarland University Medical Center, Homburg, Germany; 13 Institute of New Frontier Research, Hallym University College of Medicine, Chuncheon, South Korea; 14 Stroke Research Centre, University College London, Institute of Neurology, London, UK; 15 National Cerebral and Cardiovascular Center Hospital, 6-1 Kishibe-Shimmachi, Suita, Osaka, Japan; 16 Department of Neurosurgery, Hallym University College of Medicine, Chuncheon, South Korea; 17 Department of Pathology and Immunology, Faculty of Medicine, University of Geneva, Geneva, Switzerland; 18 Departments of Neurology, Anesthesia/Critical Care Medicine, Neurosurgery and General Internal Medicine, Johns Hopkins School of Medicine, Baltimore, MD 21287 USA

**Keywords:** Subarachnoid haemorrhage, Stroke, Outcome assessment, Health care, Genetics, Medical

## Abstract

Aneurysmal subarachnoid haemorrhage (aSAH) results in persistent clinical deficits which prevent survivors from returning to normal daily functioning. Only a small fraction of the variation in clinical outcome following aSAH is explained by known clinical, demographic and imaging variables; meaning additional unknown factors must play a key role in clinical outcome. There is a growing body of evidence that genetic variation is important in determining outcome following aSAH. Understanding genetic determinants of outcome will help to improve prognostic modelling, stratify patients in clinical trials and target novel strategies to treat this devastating disease. This protocol details a two-stage genome-wide association study to identify susceptibility loci for clinical outcome after aSAH using individual patient-level data from multiple international cohorts. Clinical outcome will be assessed using the modified Rankin Scale or Glasgow Outcome Scale at 1–24 months. The stage 1 discovery will involve meta-analysis of individual-level genotypes from different cohorts, controlling for key covariates. Based on statistical significance, supplemented by biological relevance, top single nucleotide polymorphisms will be selected for replication at stage 2. The study has national and local ethical approval. The results of this study will be rapidly communicated to clinicians, researchers and patients through open-access publication(s), presentation(s) at international conferences and via our patient and public network.

**Supplementary Information:**

The online version contains supplementary material available at 10.1007/s12975-021-00978-2.

## Introduction

Aneurysmal subarachnoid haemorrhage (aSAH) has a severe socioeconomic burden [1] as it affects people of young age and survivors suffer persisting physical, cognitive, auditory and psychosocial morbidity [2], leading to unemployment [3]. It is a striking clinical observation that individuals with similar bleeds, clinical characteristics and comorbidities experience widely different outcomes. Dozens of modelling studies, including the most recent enrolling over 10,000 patients [4], have consistently found that only up to a third of the variance in clinical outcome can be explained using a combination of demographic, clinical and imaging characteristics. Consequently, unknown additional factors play a key role in clinical outcome.

The mechanism of neurological injury following aSAH can be divided into an early brain injury (EBI) occurring within 72 h of ictus and a delayed brain injury occurring in the subsequent days to weeks after haemorrhage [5]. EBI is caused by a rapid rise in intracranial pressure and concomitant fall in cerebral blood flow at the time of haemorrhage [6]. Toxic cascades initiated by EBI and the presence of blood and its breakdown products within the cerebrospinal fluid are thought to lead to delayed brain injury, characterised by a range of pathological processes including cerebral vasospasm, inflammation, oxidative damage and cortical spreading depression [7–9]. However, the relative significance of each of these pathological pathways is currently unknown, impairing the development of pharmacological and therapeutic strategies to prevent or reduce neurological injury.

Previous studies have indicated that clinical outcome may be influenced by the genetic background of individual patients [10]. For example, we have shown that haptoglobin genotype influences clinical outcome [11] and others have implicated endothelial nitric oxide synthase [12], apolipoprotein E [13], brain-derived neurotrophic factor [14] and genes associated with fibrinolysis [15] and inflammation [16]. An automated search of the literature using GLAD4U, a PubMed gene retrieval and prioritisation tool, identified 324 genes associated with aSAH outcome [17, 18]. These genes were derived from candidate gene studies that rely on a priori knowledge of the genes to make the link with clinical outcome. However, as our understanding of the molecular mechanisms underlying outcome after aSAH is incomplete, these targeted approaches may overlook significant genes and are unlikely to deliver novel findings. A systematic genome-wide analysis will overcome this limitation. While there have been several genome-wide association studies (GWAS) comparing patients with and without aneurysms, or comparing patients with ruptured versus unruptured aneurysms, a GWAS of clinical outcome after aSAH has never been performed to date [19, 20]. This is primarily because of the logistic difficulties associated with collecting clinical outcome in a large number of patients when aSAH has a relatively low incidence of around 6 per 100,000 person-years [21]. In order to address the challenge of adequate case ascertainment, one method is to perform an individual patient level data analysis of retrospective data from multiple collaborators [22].

A greater understanding of the genetic variants associated with outcome following aSAH will provide valuable insight into the pathophysiological mechanisms of outcome after aSAH highlighting the pathways which underlie neurological injury, with the potential to improve patient care. Moreover, genetic variants could be used to improve current prognostic models identifying patients at risk of deterioration who may benefit from increased observation, early intervention or access to rehabilitation [23]. Improved prognostication will also allow patients and carers to scale their expectations and forward plan their work and personal life. Additionally, knowledge of genetic variants associated with clinical outcome could be used to adjust for patient heterogeneity in aSAH clinical trials allowing for patient stratification and decreasing required sample sizes [24]. Finally, genome-wide analysis of genetic variation associated with outcome may highlight genes and pathways that were previously not considered pharmaceutical targets to improve outcome. This will allow drug target analysis for the development of novel and/or repositioning of known therapies to mitigate the devastating consequences of this condition [25]. New treatments are desperately needed in aSAH as at present there is only a single drug (nimodipine) to improve outcome, the effect of which is modest [26, 27].

In this study protocol, we detail the methodology for the first GWAS of clinical outcome following aSAH. Our primary aim is to identify genetic variants which influence clinical outcome, irrespective of whether this is via a direct or indirect path. Consequently, genetic variants associated with outcome identified in this study may not directly influence outcome but rather mediate their effect through the pathological processes of EBI and delayed brain injury. We estimate the study power and describe the statistical methods, including quality control and adjustment for necessary covariates. This study will entail a major international collaboration to identify genes associated with clinical outcome following aSAH. This study will have three main impacts: (1) the development of improved prognostic models for aSAH outcome; (2) the identification of novel therapeutic targets; and (3) the building of the foundations of knowledge needed for future studies on clinical outcome after aSAH.

## Materials and Methods

This study will be reported in accordance with the “STrengthening the REporting of Genetic Association studies” (STREGA) statement [28]. This study was initially conceived in 2019, with patient recruitment commencing in January 2021. The authorship includes a statistician (DR) who has advised on study design and analysis.

### Study Design

International two-stage multi-centre individual patient-level data GWAS. Case-only analysis comparing good and poor outcome aSAH individuals.

### Case Ascertainment Sources

Cases for inclusion in this study are being identified from two sources:

#### Haemoglobin after intracranial haemorrhage (HATCH) consortium

The HATCH consortium is an international consortium with a focus on outcome following brain haemorrhage, including members from Asia, the Americas and Europe. The consortium is identifying adult aSAH patients by contacting investigators worldwide, identified through clinical trial registries and PubMed searches.

A trial registry search was performed (Table 1) using search conditions: “subarachnoid h(a)emorrhage” AND registration of trial in the last 10 years. The principal investigators were emailed in their native language, inviting them to participate in the study. In January 2019, 498 studies were identified, which were manually screened for relevance to biosample and/or genetic data availability, resulting in 148 contacts who were emailed. This search will be repeated prior to commencing the stage 2 validation analysis (see below) to ensure all available cases are identified.

**Table 1:** Table of trial registries searched to identify cases for inclusion in the study

Trial registry	Website
WHO International Clinical Trials Registry Platform	http://apps.who.int/trialsearch/
ClinicalTrials.gov	https://clinicaltrials.gov/
European Clinical Trials Database	https://www.clinicaltrialsregister.eu/ctr-search/search
International Standard Registered Clinical/Social Study Number (ISRCTN) registry	http://www.isrctn.com/

In collaboration with the International Stroke Genetics Consortium (ISGC), further study sites are being identified through peer networks and presentations at ISGC workshops. In addition, an international campaign, including online advertisements and multiple oral and poster presentations at several international workshops and conferences, has also been commenced.

#### The UK Biobank

The UK Biobank is an ongoing population-based cohort study that aims to improve the prevention, diagnosis and treatment of a wide range of diseases. Extensive genetic and clinical data have been collected on around half a million participants across the UK that were aged between 40 and 69 at the time of recruitment from 2006 to 2010. The design, data collection and processing are described in detail elsewhere [29]. The UK Biobank includes a substantial cohort of aSAH patients with follow-up data. The UK Biobank has approved this project proposal under application ID 49305.

### Inclusion/Exclusion Criteria

#### HATCH dataset

Adult (≥ 18 years) aSAH cases with aneurysmal cause of bleed confirmed by any angiographic method and genome-wide genotype information available will be eligible for inclusion. Individuals will be excluded if no aneurysm can be identified or if a non-aneurysmal cause for subarachnoid haemorrhage, including vascular malformation and trauma, is present.

#### UK Biobank dataset

aSAH cases will be identified from the UK Biobank using the following data fields (Supplementary Table 1):

- ICD-9 (data field 41271) and ICD-10 (data field 41270) codes from hospital inpatient data
- Read code information from primary care data (data field 42040)
- Self-reported medical conditions (data field 20002) reported at baseline or subsequent assessment centre visits

Cases identified from the UK Biobank will be cross-checked against the algorithmically generated subarachnoid haemorrhage diagnosis (data field 42012) and first occurrence database for ICD-10 code I60 (data field 131360). Genotyped aSAH cases will be included if they have outcome data available subsequent to the date of diagnosis. Cases will be excluded if there is evidence that subarachnoid haemorrhage is secondary to non-aneurysmal pathologies such as vascular malformation or trauma. Non-aneurysmal causes for subarachnoid haemorrhage will be identified using ICD-9 and ICD-10 codes indicative of non-aneurysmal SAH from hospital inpatient and primary care data (Supplementary Table 1) and individuals with such a code will be excluded regardless of the time interval between diagnosis of subarachnoid haemorrhage and potential non-aneurysmal cause. With respect to traumatic event codes, cases will only be excluded if the date of these events indicates that trauma occurred within 30 days before or after the diagnosis of subarachnoid haemorrhage.

### Primary Outcomes and Covariates

1. HATCH dataset

The primary outcome will be dichotomised clinical outcome (assessed at 1–24 months), based on the modified Rankin Scale (mRS) [30–32] or Glasgow Outcome Scale (GOS) [33, 34], which correlate highly with each other (*R*_S_ = −0.90, *p* <0.001, manuscript under review). Outcome will be dichotomised into favourable (mRS=0–2, GOS=4–5) and unfavourable (mRS=3–6, GOS=1–3), enabling both scales to be used [4]. If both mRS and GOS data are available from a single study only, the variable with the greatest data availability will be used, i.e. mixed mRS or GOS data from individual study sites will not be used.

2. UK Biobank dataset

The primary outcomes will be employment status (data field 6142) and cognition, as measured by reaction time (data field 20023) following aSAH. These measures have been chosen as they have been shown to detect differences in outcome between aSAH cases and controls within the UK Biobank cohort [35].

Employment status will be dichotomised into good and poor outcome with poor outcome defined as “unable to work because of sickness or disability” or “unemployed”. Reaction times will be ranked and then dichotomised so that the lower scoring poor outcome group is equivalent, in terms of percentage of the total UK Biobank aSAH cohort, to the poor outcome group in the HATCH dataset. Cognitive outcome and mRS after aSAH are highly correlated [36].

### Essential Covariates

**Fig. 1:**
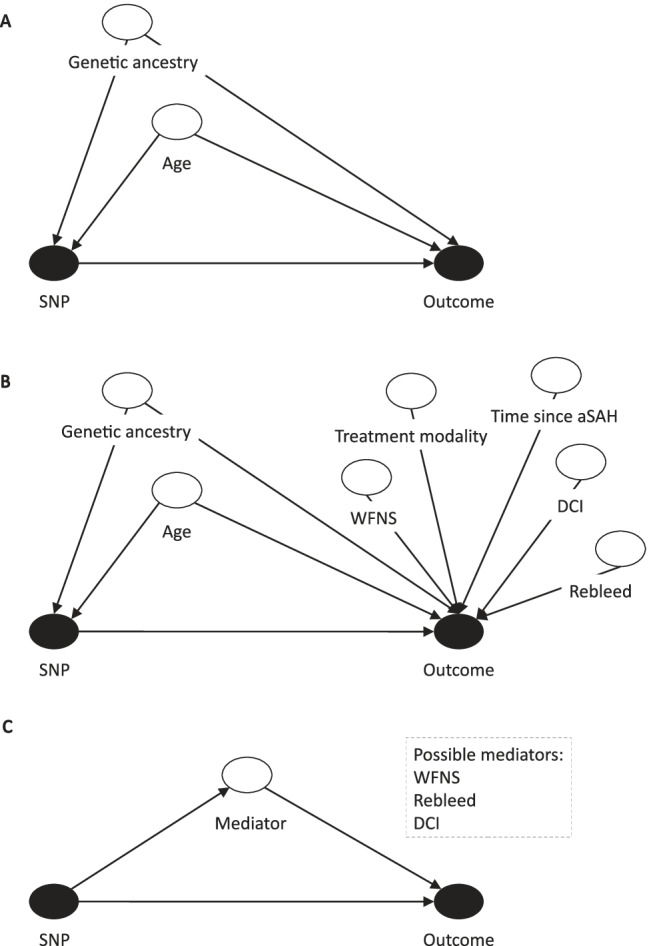
**A** Directed acyclic graph (DAG) demonstrating confounding covariate interaction with exposure (SNP) and outcome — primary analysis. **B** DAG demonstrating both confounding and selected non-confounding covariates — predictive modelling in cases with available data, for genetic variants confirmed in **A**. **C** Pathway diagram demonstrating possible mediation of a gene ➔ outcome effect by WFNS, rebleed or DCI, for genetic variants confirmed in **A**. SNP, single nucleotide polymorphism; WFNS, World Federation of Neurological Surgeons; aSAH, aneurysmal subarachnoid haemorrhage; DCI, delayed cerebral ischemia

The primary aim is not to explain maximum variance as is conventionally done in predictive modelling but to detect associations between genetic variation and outcome. We have limited covariates to confounding variables since this is essential in establishing causality; confounding variables are defined by a forward path linking the variable to both exposure and outcome. Directed acyclic graph theory has been used to rationalise the choice of covariates. Age and genetic ancestry [37, 38] are the only known variables satisfying this definition and will be included as essential covariates (Fig. 1A).

Population stratification will be assessed by principal component analysis, using reference populations from the 1000 Genomes Project [39], and the top five genetic ancestry eigenvectors will be used as covariates.

### Additional Covariates

In addition to age and genetic ancestry, a number of variables have been shown to predict outcome following aSAH. These predictors include baseline characteristics such as the World Federation of Neurological Surgeons (WFNS) score [4] and features of the patient’s clinical course, for example, aneurysm treatment modality [40], rebleed and delayed cerebral ischemia (DCI) [41]. As these variables are expected to influence outcome but not an individual’s genetic profile, they are not considered as confounding variables (Fig. 1B). However, one or more of these variables may mediate a proportion of the effect of genetic variation on outcome, and hence can be viewed as “mechanistic” variables linking gene to outcome. This is more likely for mechanistic variables with proven links to outcome such as DCI and aneurysm rebleed, both of which also happen to have a proposed genetic component [10, 19]. By focussing on the gene ➔ outcome pathway irrespective of mediating variables, this study will detect the genes that matter as potential biological targets for new treatments. In addition, this approach has the added advantage of maximising sample size since it is not dependent on the availability of additional covariates.

As an exploratory endpoint where data availability allows, regression-based mediation analysis will be used to explore whether potential mechanistic variables (WFNS score or Hunt and Hess (H&H) grade, aneurysm rebleed and presence of DCI) mediate any proportion of the genetic effect on outcome following aSAH, for genetic variants confirmed in the primary analysis (Fig. 1C). If data availability allows, an attempt will be made to construct a predictive model to explain maximum variance in outcome, including the covariates: genetic variants confirmed in the primary analysis, age, genetic ancestry, time since aSAH, WFNS score or H&H grade, treatment modality categorised as conservative, endovascular and surgical, aneurysm rebleed and presence of DCI (Fig. 1B). This will be performed as a sensitivity analysis as data is not available for all samples.

In this study, clinical outcome in the HATCH cohort is assessed over a 1- to 24-month time period. This range is broad to maximise patient inclusion in the study. The UK Biobank time to follow-up after aSAH is also broad (1 to 662 months). We have not restricted the time period over which outcome can be assessed in the UK Biobank as we have shown that cognitive and employment outcomes differ between cases and controls over this time period [35], allowing maximum patient inclusion in the study. Outcome is expected to be associated with time since aSAH (Fig. 1B) [42]. Hence, time to follow-up will be included as an additional covariate in a sensitivity analysis of significant genetic variants identified in the primary analysis. We will also conduct a sensitivity analysis including only individuals with follow-up at 3 to 6 months.

In the UK Biobank WFNS is not available, so the length of stay will be used instead since this has a strong association with WFNS [43]. As length of stay data has high missingness (around 40%) and as it is a surrogate of the strongest predictor of clinical outcome [4], it will be imputed using a method of mean imputation to allow for inclusion in the analysis. As the UK Biobank cohort uses cognitive performance and employment as surrogate measures of outcome, the additional covariates Townsend deprivation score [44] (data field 189) and education status dichotomised into individuals holding a college or university degree at the time of initial assessment in the UK Biobank or not (data field 6138) will be included in a sensitivity analysis. For the reaction time analysis, the presence of medications known to influence reaction time in the UK Biobank [45] will also be used.

### Genotype Quality Control

Where possible, single nucleotide polymorphism (SNP) data will be sought from collaborators with genotype calls relative to the positive strand. Datasets that are not genotyped on the positive strand will be identified and flipped to the positive strand using SNPFLIP. Genome-wide genotype data will be subjected to standard quality control methods. Patients with gender mismatch, individual missingness >10%, heterozygosity rates ±3 standard deviations from the samples’ heterozygosity rate mean and cryptic relatedness (proportional identity by descent > 0.1875) will be excluded. SNPs with extreme deviation from Hardy-Weinberg equilibrium, minor allele frequency (MAF) of <1% and SNP call rate <90% will be excluded. In preparation for imputation and to resolve any residual strand issues, SNPs will be excluded if they are absent from the haplotype reference consortium (HRC), their alleles disagree with HRC, their MAF differ by greater than 0.2 versus HRC or they are palindromic and have MAF greater than 0.4.

### Imputation

In the HATCH dataset, imputation will be needed since the genetic data has been obtained on different platforms; it will also increase the density of coverage to enable fine mapping around significant loci. If data has already been imputed by the contributing study teams, this imputation will be used; otherwise, imputation will be performed using the Sanger Imputation Service [46]. Haplotypes will be pre-phased using EAGLE2 [47] into the Haplotype Reference Consortium (r1.1) [46] and imputed using the positional Burrows-Wheeler transform [48]. Imputed genotypes will be quality controlled by excluding SNPs with a posterior genotype probability less than 0.8, a MAF less than 5%, greater than 10% missing genotypes within the cohort or extreme deviation from Hardy-Weinberg equilibrium (*p*≤1×10^−10^).

For datasets already quality controlled and imputed by the contributing team, the above metrics will be reapplied to ensure harmonisation with the exception of heterozygosity which relies on standard deviation of the mean and will therefore be manually reviewed, and imputation quality for which we will report the threshold for each imputed dataset.

### Data Analysis

**Fig. 2:**
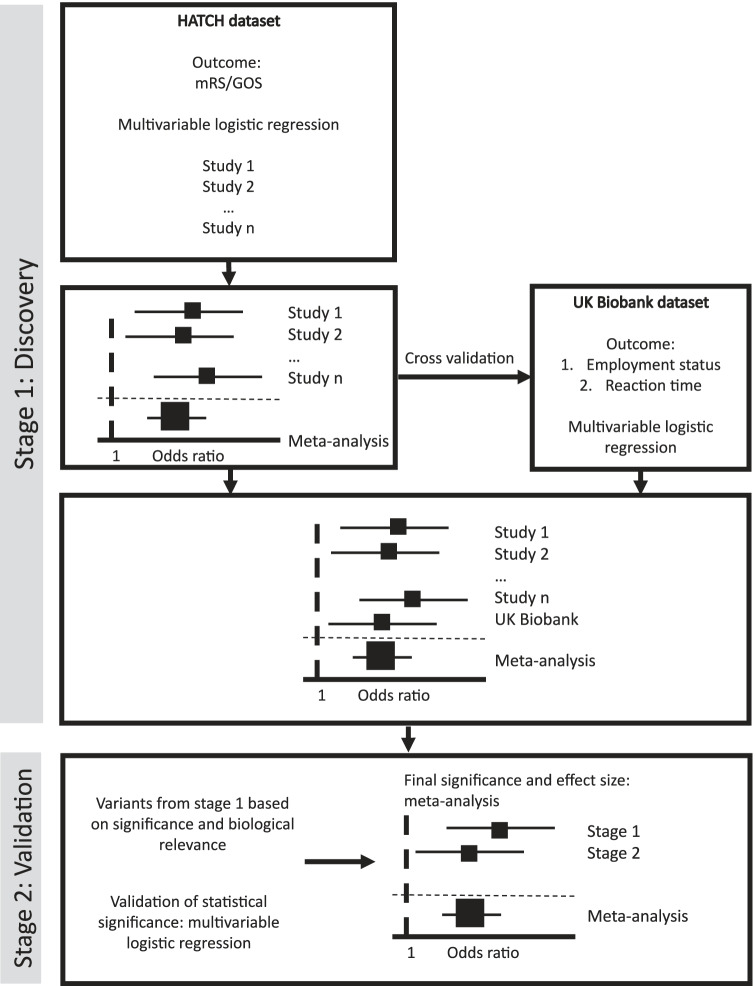
Analysis plan based on two stages: discovery and validation

The GWAS will be performed in two stages: discovery and validation (Fig. 2).

#### Stage 1: Discovery

In the first stage, available cohorts from the HATCH consortium will be analysed separately. Genetic variants will be tested for association using multivariable logistic regression analyses with dichotomised clinical outcome as dependent, genetic variant as predictor, and including the essential covariates specified for the analysis, as detailed above. Residual population stratification will be monitored using lambda from QQ plots and corrected if required. Fixed effects inverse variance weighted meta-analysis of the individual studies will be performed to determine the overall significance and effect size of individual genetic variants in the HATCH consortium. Finally, heterogeneity between cohorts will be examined using Cochrane’s *Q* and *I*^2^ statistics.

The UK Biobank will be considered a single study site and undergo the same multivariable logistic regression analysis as the HATCH dataset. Within the UK Biobank dataset, dichotomised employment status and reaction time outcomes will be considered as separate analyses. Significant findings within the HATCH dataset will be cross-validated in the UK Biobank cohort. As cognitive and traditional (mRS/GOS) outcome measures are correlated after SAH [36], a meta-analysis of summary statistics from both HATCH and UK Biobank datasets will also be performed as the final output of the stage 1 discovery analysis.

To explore the functional relevance of regions associated with clinical outcome, FUMA [49] will be used to determine if the risk SNPs and their proxies (*r*^2^≥0.6, within 1Mb and nominally significant) are located within putative functional elements such as active histone marks or transcription factor binding motifs. Additionally, FUMA will be used to annotate these SNPs with respect to evidence of regulatory function using Regulome DB scores [50], combined annotation-dependent depletion scores [51] and gene expression using eQTL analysis [52]. Furthermore, genes functionally related to the risk SNPs will be explored as drug targets using individual gene and network-based approaches. Finally, phenome-wide association study techniques will be used to identify SNPs with pleiotropic effects.

#### Stage 2: Validation

Genetic variants with the greatest significance in stage 1 will be identified for replication in the stage 2 validation analysis. Variants from stage 1 will be selected for stage 2 based on statistical significance, with all variants with *p*<1×10^−4^ considered for replication. The top variants, as ranked by *p*-value, will be included in the validation study. In addition, to maximise the identification of replicable variants in stage 2 [53], variants from stage 1 with *p*<1×10^−4^ and evidence of biological/functional relevance will also be included in the validation study. The validation study will use the same multivariable logistic regression analysis as stage 1. Only variants that replicate in the validation study will be considered to be truly associated with outcome with the final significance and effect size determined by a fixed effects meta-analysis of stages 1 and 2.

### Sensitivity Analyses

In order to account for time since aSAH, it will be included as an additional covariate with significant genetic variants from the primary analysis retested, incorporating this variable to ensure an independent genetic effect (Fig. 1B). In addition, a further sensitivity analysis will be performed, including only individuals with follow-up at 3 to 6 months.

As the UK Biobank cohort uses employment and cognition as surrogate measures of outcome, a sensitivity analysis including the additional covariates described above will be performed to ensure independent genetic effect. Finally, the UK Biobank cohort will be excluded to ensure no change to the significance of the results.

### Mediation Analyses

Regression-based mediation analysis will be used to explore whether potential mechanistic variables (WFNS score or H&H grade, aneurysm rebleed and presence of DCI) mediate any proportion of the genetic effect on outcome following aSAH, for genetic variants confirmed in the primary analysis.

### Sample Size and Power Calculation

A recent study demonstrated that in high Fisher grade (III–IV) individuals, the haptoglobin 2-2 genotype was associated with good clinical outcome (mRS 0–1) following aSAH with an odds ratio of 2.6 (95% confidence interval 1.4–4.9) [11]. Based on this finding, we aim to power this study to detect common genetic variation with an effect size of >1.4, the lower end of the 95% confidence interval. After aSAH, 30% are expected to have an unfavourable outcome [54].

At present, approximately 2500 retrospective samples have been identified for inclusion in the stage 1 (discovery) analysis. See www.southampton.ac.uk/hatch/studies/gwas.page for a live tally of sample number and study sites. Recruitment is ongoing for the stage 2 (validation) study with multiple international collaborators. The co-authors of this study have either provided data for stage 1 or are providing samples or data for the stage 2 analysis.

**Fig. 3:**
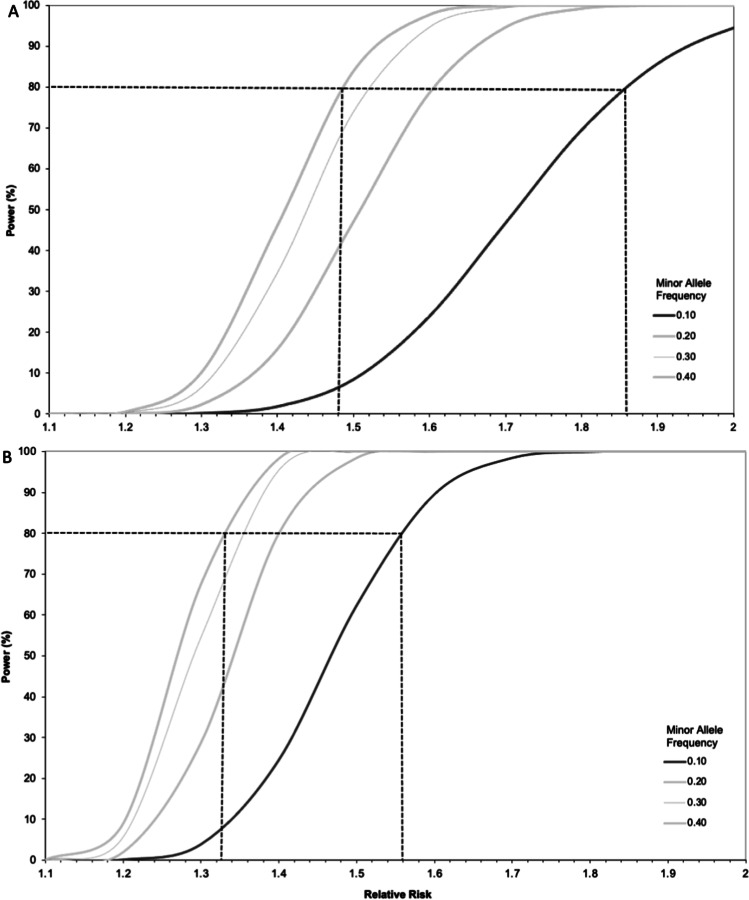
Graph of power versus SNP effect size for a range of minor allele frequencies at genome-wide significance. Dashed line identifies effect size at 80% power. **A** Sample size = 2500 (stage 1); **B** sample size = 5000 (final meta-analysis: stage 1 + stage 2)

Based on the current stage 1 discovery analysis sample size of 2500, according to the above event rate and assuming an additive model, the stage 1 analysis will have 80% power to detect common SNPs (MAF=0.4) with an effect size of 1.48 and rare SNPs (MAF=0.1) with an effect size of 1.86 at a genome-wide level of significance (Fig. 3A). In the final meta-analysis combining stage 1 and stage 2, a sample size of 5000 is predicted, which using the same assumptions will have 80% power to detect common SNPs (MAF=0.4) with an effect size of 1.33 and rare SNPs (MAF=0.1) with an effect size of 1.56 at a genome-wide level of significance (Fig. 3B).

### Limitations

This study does not include individuals who died prior to hospital admission. In addition, it is possible that recruitment is biased towards better outcome individuals as poor prognosis individuals who die in the first few days after aSAH are less likely to be recruited. We will assess this bias by comparing the proportion of good/poor outcome patients in this study with that in contemporary observational studies of outcome and case fatality [55]. Even if such a bias is identified, it will not be a significant limitation to this study as the ultimate aim is to identify pathophysiological mechanisms which can act as therapeutic targets to improve outcome. After aSAH, the time window over which individuals deteriorate following a bleed is relatively long (days to weeks) and during this time patients usually remain in hospital. This means that there is a window of opportunity to administer treatments to prevent deterioration and poor outcome following haemorrhage. Unfortunately, individuals who die prior to admission or in the first few days after aSAH are unlikely to benefit from such interventions. This means that although our study population may be biased away from individuals who die in the first few days after aSAH, it includes the individuals who realistically will benefit from the output of this study.

The UK Biobank has different outcome measures (cognition and employment) than the HATCH datasets that employ the mRS or GOS. This limits the comparability of the UK Biobank and HATCH datasets. To address this limitation, we have separated the UK Biobank and HATCH datasets, and the UK Biobank will be used to cross-validate findings from the HATCH datasets. This allows significant findings in the HATCH data to be further validated using outcome metrics more sensitive to the nuances of outcome and more relevant to patients than mRS/GOS. We also combine the UK Biobank and HATCH datasets to maximise study power. Within the UK Biobank, only 2% of individuals have psychomotor reaction time and/or employment status recorded both before and after aSAH. Nevertheless, a detailed analysis of employment and cognition in the UK Biobank has shown that psychomotor reaction time and employment are significantly impaired compared to matched controls and therefore constitute valid outcome measures [35].

### Patients and Public Involvement

We have worked with the Wessex Subarachnoid Haemorrhage Support Group and participants from previous research studies of patients with aSAH to prioritise research questions important to them and their carers since 2012, holding regular meetings and workshops. The group identified maximising use of samples obtained from prior studies as an important principle and understanding mechanisms of poor outcome to develop new treatments as a priority.

### Ethics

For this study, national (REC 19 SC 0485) and local (ERGO 49253) ethical approvals are already in place.

### Dissemination

The output of this discovery study will be published in relevant open-access peer-reviewed journals to ensure rapid dissemination to the target audience. All contributors to the study will be co-authors on manuscripts alphabetised between first and senior authors. Results of the study will also be presented at national and international stroke and aSAH meetings. In addition, through our links with the Wessex SAH support group, we will promote the output of this study to patients and the public along with presentation of results on the HATCH, local hospital and university websites, and social media.

## Supplementary Information

ESM 1 (PDF 69 kb)

## Data Availability

Study data will be available from the authors subject to institutional agreements and ethical approvals.
