# Supplementary material for: Genome-Wide Association Study of Clinical Outcome After Aneurysmal Subarachnoid Haemorrhage: Protocol
Source: Transl Stroke Res. 2022 Jan 6;13(4):565–76. doi: 10.1007/s12975-021-00978-2 (PMC9232474; doi:10.1007/s12975-021-00978-2)
Supplement: Supplementary file 1 — (PDF 69 kb) [file 12975_2021_978_MOESM1_ESM.pdf]

Supplementary information

Genome-wide association study of clinical outcome after aneurysmal subarachnoid haemorrhage: a protocol

Translational Stroke Research

Ben Gaastra, Sheila Alexander, Mark K. Bakker, Hemant Bhagat, Philippe Bijlenga, Spiros Blackburn, Sylvain Doré, Malie K. Collins, Christoph Griessenauer, Philipp Hendrix, Eun Pyo Hong, Isabel C. Hostettler, Henry Houlden, Koji IIhara, Jin Pyeong Jeon, Bong Jun Kim, Munish Kumar, Sandrine Morel, PA Nyquist, Dianxu Ren, Ynte M. Ruigrok, David Werring, Ian Galea*, Diederik Bulters, Will Tapper

*corresponding author email: i.galea@soton.ac.uk

Data field	Code and definition
Inclusion	
Data field 41270<br>ICD 10 codes	I600 I60.0 Subarachnoid haemorrhage from carotid siphon and bifurcation<br><br>I601 I60.1 Subarachnoid haemorrhage from middle cerebral artery<br><br>I602 I60.2 Subarachnoid haemorrhage from anterior communicating artery<br><br>I603 I60.3 Subarachnoid haemorrhage from posterior communicating artery<br><br>I604 I60.4 Subarachnoid haemorrhage from basilar artery<br><br>I605 I60.5 Subarachnoid haemorrhage from vertebral artery<br><br>I606 I60.6 Subarachnoid haemorrhage from other intracranial arteries<br><br>I607 I60.7 Subarachnoid haemorrhage from intracranial artery, unspecified<br><br>I609 I60.9 Subarachnoid haemorrhage, unspecified<br>Ruptured aneurysm
Data field 41271<br>ICD9 codes	430 Subarachnoid haemorrhage<br><br>4309 Subarachnoid haemorrhage
Data field 42040<br>Primary care data	G60 Equates to ICD-10 code I609<br><br>G600 Equates to ICD-10 code I607

	G601 Equates to ICD-10 code I600<br><br>G602 Equates to ICD-10 code I601<br><br>G603 Equates to ICD-10 code I602<br><br>G605 Equates to ICD-10 code I604<br><br>G606 Equates to ICD-10 code I605<br><br>G60X Equates to ICD-10 code I607<br><br>G60z Equates to ICD-10 code I609<br><br>Gyu60 Equates to ICD-10 code I606<br><br>Gyu6E Equates to ICD-10 code I607<br><br>X00Df Equates to ICD-9 code 430<br><br>X00Dg Equates to ICD-10 code I609<br><br>X204F Equates to ICD-10 code I609<br><br>Xa01c Equates to ICD-10 code I606<br><br>Xa01h Equates to ICD-10 code I601<br><br>Xa01i Equates to ICD-10 code I606<br><br>Xa01j Equates to ICD-10 code I602<br><br>Xa01k Equates to ICD-10 code I603<br><br>Xa01l Equates to ICD-10 code I604<br><br>Xa01m Equates to ICD-10 code I606<br><br>Xa01o Equates to ICD-9 code 430
Data field 20002<br>Self-reported medical conditions	1086 Subarachnoid haemorrhage
Exclusion	
Data field 41270<br>ICD 10 codes	Q282 Q28.2 Arteriovenous malformation of cerebral vessels<br><br>Q283 Q28.3 Other malformations of cerebral vessels

	S-T Injury, poisoning and certain other consequences of external causes<br><br>V,W,X External causes of morbidity and mortality
Data field 41271<br>ICD9 codes	74780 Arteriovenous aneurysm of brain<br><br>74781 Other anomalies of cerebral vessels<br><br>800-900 Trauma and injury
Data field 42040<br>Primary care data	P7y01 Equates to ICD-10 code Q282<br><br>P7y02 Equates to ICD-10 code Q283<br><br>S, U Equates to ICD-10 codes S,T,V,W,X and ICD-9 codes 800-900

Supplementary Table 1. Inclusion and exclusion codes for aSAH cases in the UK Biobank. ICD: International Classification of Diseases.
